# Draft genome of the marine bacterium *Alteromonas gracilis* strain J4 isolated from the green coenocytic alga *Caulerpa prolifera*

**DOI:** 10.1128/mra.00184-24

**Published:** 2024-06-11

**Authors:** Hannah J. van Duijnhoven, Nina Dombrowski, Peter Kuperus, Tânia Aires, Márcio A.G. Coelho, João Silva, Gerard Muyzer, Aschwin H. Engelen

**Affiliations:** 1 Centre of Marine Sciences (CCMAR/CIMAR LA), Universidade do Algarve, Campus de Gambelas, Faro, Portugal; 2 Microbial Systems Ecology, Department of Freshwater and Marine Ecology, Institute for Biodiversity and Ecosystem Dynamics, University of Amsterdam, Amsterdam, the Netherlands; Montana State University, Bozeman, Montana, USA

**Keywords:** plant-microbe interactions, whole-genome sequencing, marine microbiology, growth-promoting bacteria, interspecific competition

## Abstract

Here, we present the draft genome sequence of *Alteromonas gracilis* strain J4, isolated from the green macroalga *Caulerpa prolifera*. The draft genome is 4,492,914 bp in size and contains 4,719 coding DNA sequences, 67 tRNAs, and 16 rRNA-coding genes. Strain J4 may exhibit host growth-promoting properties.

## ANNOUNCEMENT

To understand *Caulerpa* host-microbe interaction(1,2), we isolated bacteria from *C. prolifera* rhizoid tissue and conducted whole-genome sequencing. Here, we present the draft genome sequence of *Alteromonas gracilis* strain J4.

Strain J4 was isolated from rhizoids of hand-collected *C. prolifera* in the Ria Formosa lagoon (37°00′22.7″N 7°58′00.3″W, Faro, Portugal) and stored in a cooling box. Rhizoids were ground with mortar and pestle, and the lysate was plated on Difco Marine Agar 2216 and incubated in the dark at room temperature (20–25°C). After 3 days, individual colonies were replated and re-incubated. Isolate J4 was identified as an *Alteromonas* sp. by comparative full-length 16S rRNA gene Sanger sequencing (Applied BioSystems 3130*xl* Genetic Analyzer) analysis using primers 27F/1492R against the NCBI database (3). The 16S rRNA gene showed 96.80% sequence identity to *A. gracilis* strain 9a2 341. Genomic DNA was extracted using the peqGOLD Bacterial DNA Mini Kit (VWR). Genome sequencing was conducted on a MinION Mk1C device (Oxford Nanopore Technologies), using the Ligation Sequencing Kit (SQK-LSK110) and a Flongle Flow Cell (R9.4.1). Base-calling was performed using Guppy v6.2.7 (https://community.nanoporetech.com/downloads). 225,773 reads passed quality control with a mean Q-score of 12 and an N50 of 3.71 kb.

**Fig 1:**
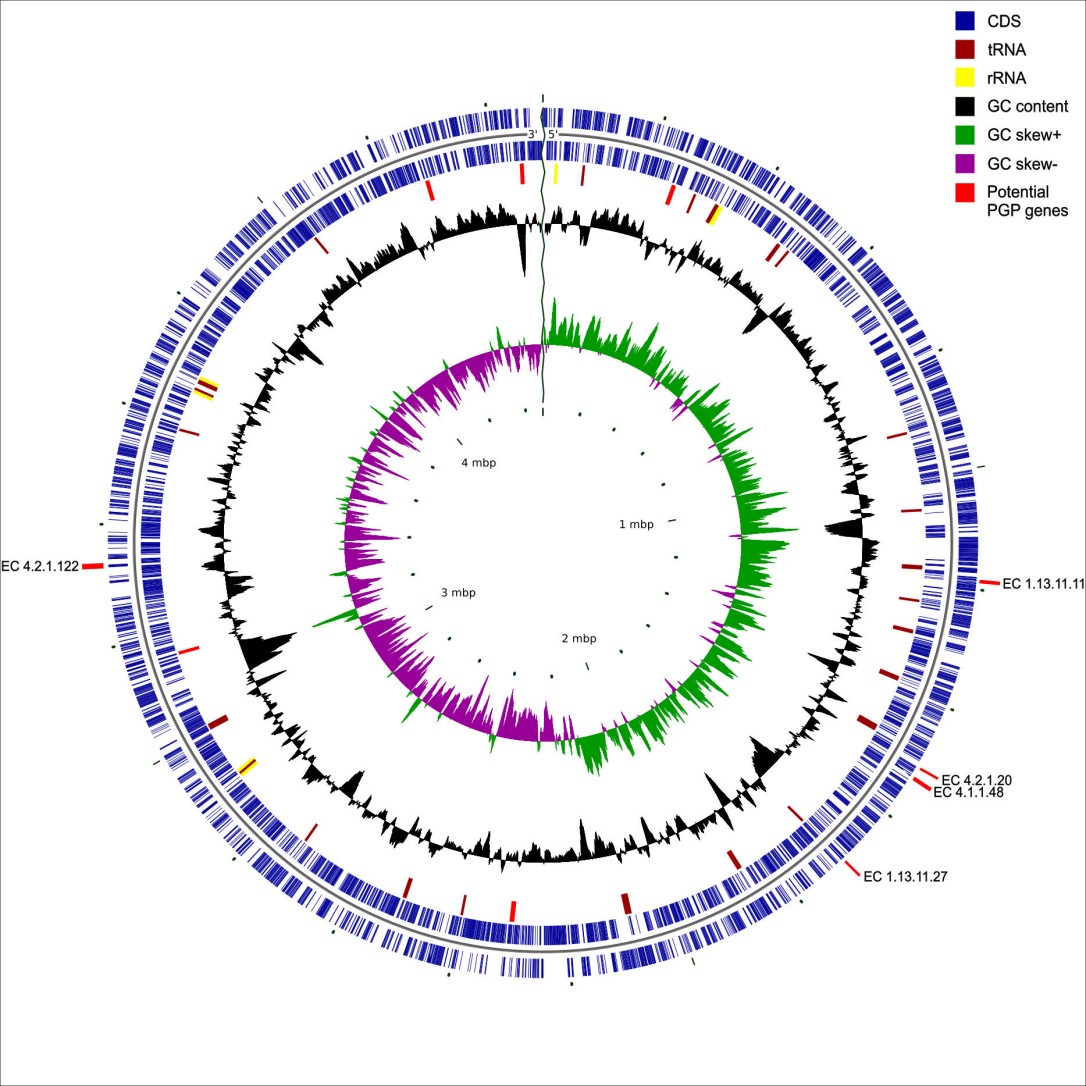
The genome map of *Alteromonas gracilis* strain J4. Each circle from inner to outer indicates potential plant growth-promoting (PGP) genes, coding sequences (CDS) in the leading strand, CDS in the lagging strand, tRNA and rRNA, GC content, GC skew+, and GC skew−. Potential PGP genes are indicated in red and labeled with EC numbers. If not labeled, tryptophan halogenases are represented.

Trimming residual sequencing adaptors and splitting chimeric reads [Porechop v0.2.4 (4)] resulted in 211,623 reads. Filtlong v0.2.1 was used to remove small (<1,000 bp) and poor quality (<5%) reads (https://github.com/rrwick/Filtlong). For the remaining 172,018 reads, 12 subsamples were generated at 55× depth using Trycycler v0.5.5 (5). Three subsamples were each assembled with (i) Flye v2.9.3, (ii) Miniasm v0.3 & Minipolish v0.1.3, (iii) Raven v1.8.3, and (iv) Unicycler v0.5.0 (6–10). The consensus assembly was further generated using Trycycler v0.5.5 and polished with Homopolish v0.4.1 (11), resulting in one circular contig with a total size of 4,492,914 bp (131× coverage) and a GC content of 44.0%. The genome was reoriented using Dnaapler chromosome v0.7.0 (12) by identifying dnaA as the replication initiator gene. The genome had a completeness of 98% and contamination of 5.1% (CheckM2 v1.0.2) (13) and contained 4,719 protein-coding genes, 67 tRNA, and 16 rRNA coding genes [Prokka v1.14.6 (14); Fig. 1]. 903 genes were identified as potential pseudogenes (https://github.com/ndombrowski/j4_assembly) using Pseudofinder v1.1.0 with the UniProtKB/Swiss-Prot database as reference (15, 16). J4 likely belongs to an uncharacterized species within the genus *Alteromonas*. The genome exhibits 85.3% average nucleotide identity (ANI) with *Alteromonas* sp009811495 (GCF_016756315.1) based on a comparison with the GTDB r214 database using GTDB-Tk v2.3.2 (17, 18).

The annotation analysis identified two genes encoding 4-hydroxyphenylpyruvate dioxygenase (EC 1.13.11.27), pivotal in melanin catalysis (19). Melanin plays a role in ensuring survival during symbiotic interactions (20). Protein genes *cspD*, *dnaJ, dnaK,* and *grpE*, described to protect against cold/heat and oxidative stress, were detected (21), as well as 12 genes related to sulfur metabolism (22). Four indole-3-glycerol phosphate synthases (EC 4.1.1.48) and two tryptophan 2,3-dioxygenases (EC 1.13.11.11), both key precursors in indole-3-acetic acid biosynthesis were found. In all, 24 putative genes encoding tryptophan halogenases and six tryptophan synthases were found, suggesting potential growth-promoting properties in strain J4 with biotechnological applications (23, 24).

## Data Availability

The 16S rRNA sequence data, raw Nanopore sequence reads, and the assembled genome sequence have been deposited in GenBank under BioProject number PRJNA1077798, with BioSample accession numbers PP541516, SAMN40604929, and SAMN39982826, respectively, and the reported genome is the second version, CP145482.2.
